# Increase and Prevalence of Typhoid Fever in Western New York

**Published:** 1865-09

**Authors:** 


### Editorial Department.

### Increase and Prevalence of Typhoid Fever in Western New York.

Some of the younger members of the profession may not be aware of the fact, that the Typhoid Fever, which is now so widely prevalent, is comparatively a new disease in Western New York; though it has been observed and recognized in rare instances ever since about the time when Louis ascertained that the fevers of Paris were related to each other by lesions of the glands of Peyer, and by a softened condition of the spleen. He first applied the term *typhoid* to distinguish a febrile disease which he had demonstrated as dependent upon this local lesion. After the publication of the researches made in France, the physicians of other countries became interested to determine how far the same form of fever prevailed elsewhere. In England it was soon found that in some cases of their so-called typhus fever, the descriptions of Louis were applicable, while in others they were not. In our own country—in New England, the indiginous or autumnal fever, was found to present symptoms and lesions entirely similar to those observed in France.

Physicians in Philadelphia observed a fever which was endemic in that vicinity, identical with the typhoid of Louis. From this beginning typhoid fever commenced to be recognized as a distinct disease, though not accepted by many pathologists as such. The British physicians more generally regarded it as a modification of continued fever, which might be divided into typhus and typhoid; while almost everywhere was acknowledged the fact that a wide distinction in these two forms of disease did actually exist.

In this vicinity the prevailing type of fever was the common remittent, which was at that time often called "bilious fever," though the editor of the Buffalo Medical Journal, Prof. Austin Flint, says, "there is reason to suppose that sporadic cases of typhoid fever occasionally occur, and we are liable at any time to meet with this, or undoubted typhus in an epidemic form." On account of this liability he published the "characters distinguishing remittent and typhoid fever."

From this and other data, we infer that typhoid fever, as such, was not a disease of Western New York, and except in rare instances had not been observed previous to 1846. Dr. G. N. Burwell in a communication to the Buffalo Medical Journal dated November 12, 1845, says, "I saw a woman on Grand Island in December last whom I considered as having typhoid fever. In June I went to Hamburgh to see a woman who undoubtedly had this fever; and in April last my father went to the same town to see a case of this disease."

Twenty years ago the autumnal fevers of New England were as truly typhoid as they are to-day, while in Buffalo the disease was of rare occurrence, and perhaps not in an unmixed form in any instance, being changed by the malarious influence which was modifying almost all forms of disease.

Gradually typhoid fever has been increasing in frequency until it is now the prevailing type of fever, while remittent fever and other forms of malarious disease have been gradually growing less frequent, and in many parts have entirely disappeared.

It would be interesting to follow inquiry into the causes of this increase in the frequency of typhoid disease, and disappearance of the former characteristic type of fever. Perhaps explanation might be readily offered of the disappearance of malarious disease, while greater difficulty would be met in accounting for the great increase in the frequency of typhoid fever. It should be remarked that in the hill towns of Erie County malarious disease was never frequent. Dr. Emmons, one of the oldest and most experienced physicians in Western New York informs us that, intermittent fever was never generated in Springville, that formerly, all disease was inflammatory in character, and all inflammations of the active type.

Typhoid fever is becoming the fever of the country, and is now occurring more frequently than ever before; in Buffalo certainly it is prevailing very extensively, and we believe it has gradually gained in frequency since its first appearance.

A proper understanding of its natural history and pathology will naturally lead to very correct modes of treatment, though it must be confessed that all plans of medication have been proposed and adopted, some cases recovering, even under the most il l directed efforts. Great uniformity now prevails in the treatment of this disease, and we have nothing to suggest which will add to the generally accepted doctrines concerning it. We have recently visited day after day and carefully watched the progress of the disease without prescribing any medicine at all, and the cases terminated early and satisfactorily. The severe symptoms which are usually present, can be more or less relieved by judicious medication, always keeping in view, that if we cannot do any good, we can at least, avoid doing harm. There is one point in the treatment of typhoid fever, to which it might be well to direct attention. Does the malarious influence which pervades this vicinity to greater or less extent, so modify or complicate all disease, typhoid fever included, that the specific or anti-periodic properties of bark are useful in its treatment? Will quinine relieve any remittent symptoms, abate complications, or in any other way favorably influence the termination of typhoid fever?

